# A negative feedback regulatory loop between miR-138 and TP53 is mediated by USP10

**DOI:** 10.18632/oncotarget.27275

**Published:** 2019-10-29

**Authors:** Zhenghua Luo, Manchao Zhang, Ri Cui, Esmerina Tili, Taewan Kim, Tae Jin Lee, Yong Peng, Carlo Croce

**Affiliations:** ^1^ Department of Cancer Biology and Medical Genetics, Comprehensive Cancer Center, The Ohio State University, Columbus, OH 43210, USA; ^2^ State Key Laboratory of Biotherapy, West China Hospital, Sichuan University, Chengdu, Sichuan 610041, P.R. China

**Keywords:** TP53, USP10, miR-138

## Abstract

TP53 is a critical tumor suppressor. In approximately 50% of human cancers the TP53 gene is either lost or mutated. The expression level of TP53 in the cells is tightly controlled by a fine-tune machinery, mainly through the Mdm2-mediated ubiquitination pathway. On the other side, the ubiquitinated TP53 could be reversed and stabilized by USP7 and USP10, to keep the amount of TP53 in check. MicroRNAs can negatively regulate TP53 expression levels through direct targeting or positively regulate TP53 function through the repression of negative regulators of TP53. Here we report that microRNA-138 controls TP53 expression by directly targeting USP10. Furthermore, TP53 represses microRNA-138 expression, forming a negative feedback regulatory loop. This finding adds another layer of complexity to the TP53 network.

## INTRODUCTION

The TP53 is one of the best-known tumor suppressor genes and it is frequently mutated in human tumors [1–3]. TP53 associates hundreds of other genes and their products to play a central role in genomic maintenance and tumor suppression [3]. Since its discovery around 30 years ago, TP53 and its signaling pathway have been extensively studied, and, at the same time, the TP53 network keeps growing in complexity. In unstressed cells, the TP53 protein levels are kept relatively low through the Mdm2-mediated ubiquitin-proteasome degradation pathway [4–6]. ATM (ataxia-telangiectasia mutated) phosphorylates Mdm2 and other members that drive TP53 ubiquitination and induces their degradation by reducing their interaction with USP7/HAUSP, thereby stabilizing TP53 when cells encounter stress [7–9]. USP10 directly deubiquitinate TP53 so that the TP53 levels were further increased [10]. In roughly 50% of human cancers, TP53 is lost or mutated. Interestingly, many of these mutations give birth to highly expressing of mutant TP53 proteins [11–13]. Various lines of evidence indicate that, in addition to lose the tumor suppressor function, the mutant TP53 protein might contribute actively to tumor progression and to increased resistance to anticancer drug treatments [14, 15]. USP10 has been found to be able to stabilize both wild-type and mutant TP53, suggesting it works as either a tumor suppressor or an oncoprotein [10].

microRNAs (miRNAs) are a class of endogenously expressed 19-25 nucleotides long non-coding regulatory RNA molecules [16]. In general, microRNAs bind to the 3′-untranslated regions (3′-UTR) of messenger RNA (mRNA) in a sequence specific manner, thus induce mRNA degradation or inhibit translation [17]. To date, more than 2,500 human miRNA candidates have been recorded and the number keeps increasing rapidly. In different cell contexts or development stages the expression of miRNAs could be different. miRNAs can function as oncogenes or tumor suppressor genes during tumor development and progression in human cancers [18]. Many miRNA signatures are available now to accurately distinguish tumor from healthy tissue. miRNAs can serve as candidate biomarkers or diagnostic and prognostic purposes [18].

TP53 has been recently found to be closely interacting with miRNAs [19]. TP53 positively induces the expression of several miRNAs [19, 20]. Some miRNAs directly target TP53 and negatively regulate TP53 levels and its function [21, 22]. In other cases, miRNAs can positively regulate TP53 activity by down-regulating negative regulators of TP53 [23]. These findings add another layer of complexity to the TP53 network.

miR-138 has been broadly studied in human cancers. It has been reported that miR-138 induces cell cycle arrest in hepatocellular carcinoma (HCC) [24]. miR-138 promotes apoptosis and suppresses invasion in head and neck squamous cell carcinoma (HNSCC) cell lines [25]. These studies indicate that miR-138 probably serves as a tumor suppressor. Interestingly, in HNSCC patients, the chance of TP53 mutation could be up to 90% [26]. And around 70% of HCC patients carry a mutated form of TP53 [27]. miR-138 has also been suggested to serve as an oncogene in gliomas [28]. Whether or not miR-138 plays a role in regulating p53 is unknown.

In this study, we identified miRNA-138 directly binds to the 3′-UTR of USP10 mRNA, whose product is a positive regulator of TP53. We found that miRNA-138 represses USP10 expression to down-regulate TP53 protein levels, leading to a decrease in TP53 function, reduction of cell apoptosis and defection in cell cycle arrest. Stable cell lines expressing miRNA-138 promotes the growth of xenograft tumors in mice. In addition, we found TP53 could repress miRNA-138 expression by direct binding to the miRNA-138 promoter regions, implying miRNA-138 and TP53 form a negative regulatory loop. Notably, as USP10 can positively regulate both wild-type and mutant TP53, therefore, miR-138 could also play a dual role as an either tumor suppressor or an onco-miR in different cell context. This finding highlights miR-138 could be a potential therapeutic target for the 50% of human cancers that express mutated TP53.

## RESULTS

### miR-138 targets USP10

**Figure 1:**
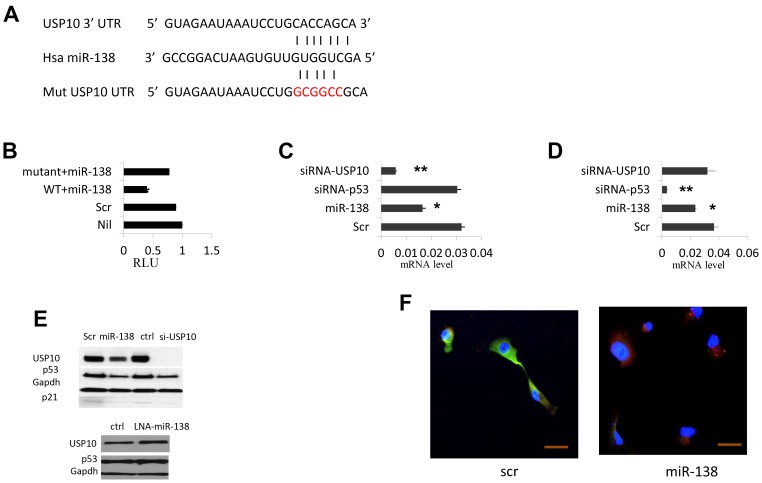
miR-138 regulate TP53 expression by targeting USP10. (**A**) USP10 3′-UTR. fragment harboring the putative miR-138 binding site. Seed sequences of miR-138 match to USP10 are shown with bars. Site-directed mutagenesis to abolish miR-138 targeting is shown in red color. (**B**) Relative luciferase (RLU) reporter assay to determine the specific targeting of miR-138 to USP10. 3′-UTR of USP10 is fused to the luciferase gene in the pGL3 vector and co-transfected with miR-138 precursor or a miRNA scramble control. Nil, no miR-138 precursor; Scr, scramble control; Wt+miR-138, wild-type 3′UTR co-transfected with miR-138; mutant, mutated form of 3′ UTR co-transfected with miR-138 precursor. (**C**) Real-time PCR USP10 mRNA accumulation levels (log scale). (**D**) Real-time PCR TP53 expression levels (log scale). Scr, scramble control; miR-138, cells transfected with miR-138 precursor; siRNA-TP53, cells transfected a siRNA against TP53; siRNA-USP10, a siRNA targeting USP10 was introduced into cells. (**E**) Above, western blotting of USP10, TP53 p21 in cells transfected scramble miRNA control or miR-138 precursor, a siRNA control or siRNA against USP10; bottom, USP10 and TP53 mRNA levels in cells transfected LNA-miR-138. GAPDH is used as an internal control. (**F**) Immunofluorescence of USP10 and TP53 in HeLa cell overexpressed miRNA-138. 24hrs after transfection cells were stained with respective antibody and live cells analyzed by confocal microscopy. Red, USP10; Green, TP53; Blue is DAPI staining of cell nuclei. >10 fields were visualized and the represents were shown. Bar 20 μm. ^*^ *P* < 0.05, ^**^ *P* < 0.005.

miR-138 works in cancer cells either as an oncogenic or tumor suppressor microRNA; USP10 regulates p53, it would be of great interest to investigate if there is a link between miR-138 and USP10 expression. Using *in silico* prediction programs, we found USP10 is a putative target of miR-138. The 3′ untranslated region (UTR) of USP10 harbors a complementary sequence of miR-138, and this fragment is highly conserved in mammals Supplementary Figure 1. To validate that USP10 is a direct target of miR-138, we constructed part of its 3′-UTR into the pGL3 vector downstream of a luciferase gene. In the meantime, we made site-directed mutagenesis in the putative seed sequence of miR-138 binding region using QuickChange Mutagenesis kit to determine the target specificity (Figure 1A). We then co-transfected HeLa cells (wild-type p53) with these constructs and miR-138 precursor and the luciferase activities were examined 48 hrs later. We found the luciferase activity was decreased about 70% in cells transfected with wild-type USP10 3′-UTR and miR-138 (*p* < 0.05, *n* = 12). However, no significant changes in the cells expressed the mutated form of USP10 3′-UTR and miR-138 (*p* > 0.05, *n* = 12. Figure 1B). These data indicate miR-138 indeed down-regulate USP10 3′ UTR, and this regulation is sequence-specific. Next, we measured the USP10 mRNA levels by realtime PCR. In cells transfected with miR-138, we observed a 2-fold decrease of the USP10 mRNA level (*p* < 0.05, *n* = 12). USP10 mRNA level was not changed in cells transfected a siRNA targeting TP53 (*p* > 0.05, *n* = 12. Figure 1C), suggesting miRNA-138 represses USP10 expression by down-regulating its transcription, while repressing TP53 does not have significant effects on USP10 expression.

### miR-138 regulates TP53 expression and its function

Previous report showed that USP10 positively regulate TP53. Since we found USP10 is a target of miR-138, we sought to decipher whether miR-138 is involved in the TP53 network through USP10. Indeed, in cells transfected by miR-138, we observed that TP53 mRNA level was reduced ~30% (*p <* 0.05, *n* = 12 Figure 1D). Western blotting also showed that p53 was reduced dramatically by miR-138 overexpressing, along with the decreased USP10 level (Figure 1E). In contrast, cells transfected a Locked-nucleic acid against miR-138 (LNA-miR-138) or miR-138 inhibitor, the TP53 mRNA level was clearly increased (Figure 1E). We also observed that both TP53 and USP10 protein levels were reduced by miR-138, as shown by diminished immunofluorescence (Figure 1F). This finding suggests that miR-138 expression resulted USP10 down-regulation lead to decreased expression of TP53.

**Figure 2:**
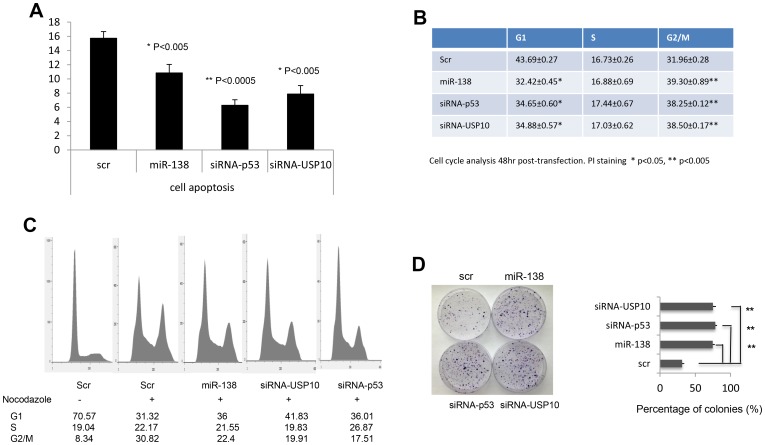
Effects of miR-138 on TP53 function. (**A**) HCT116 cells were fixed and stained with Annexin V and analyzed on FACSCalibur to determine the cell apoptosis status. (**B**) HCT116 cells were starved, fixed and stained with PI and analyzed on FACSCalibur to determine the cell cycle arrest. Scr, scramble control; miR-138, cells transfected with miR-138 precursor; siRNA-TP53, cells transfected a siRNA against TP53; siRNA-USP10, a siRNA targeting USP10 was introduced into cells. (**C**) Cell cycle arrest assay. Cells were grown in 24-well plates then treated with scramble or mir-138 or siRNAs against USP10 or p53 24 hrs later. Another 24 hrs later, cells were treated with nocodazole overnight then harvest for cell cycle analysis. (**D**) Colony formation assay. HCT116 cells transfected with scramble or miR138 or siRNAs against p53 or USP10 were seeded at low density and grown for one week. Colonies were visualized by crystal violet staining. ^*^ *P* < 0.05, ^**^ *P* < 0.005.

miR-138 regulates TP53 expression pointed the possibility that miR-138 affects TP53-dependent transcriptional activity, cell cycle, and apoptosis. As shown in Figure 1E, we observed that p21, a cell cycle regulator which transcription is directly regulated by p53, was down-regulated in cells transfected with miR-138 or USP10 siRNA, suggesting that miRNA-138 represses TP53-dependent transcriptional activity. Next, we tested whether miR-138 regulates TP53 function in modulating apoptosis and cell cycle. As shown in Figure 2A, cell apoptosis was strongly inhibited when miR-138 was overexpressed as well as when USP10 or TP53 was silenced with USP 10 siRNA or TP53 siRNA. Furthermore, overexpressing miR-138 resulted in cell cycle arrest on G2/M phase (Figure 2B), which is consistent to the results when USP10 or TP53 was down-regulated directly by its respective siRNA. In Figure 2C we show that G2/M transition was arrested with nocodazole treatment when miR-138, siRNAs against USP10 or p53 was introduced into the cells. In addition, cells with downregulated miR-138 had elevated colony formation as those transfected with p53 or USP10 siRNAs (Figure 2D). This data indicates miR-138 suppresses P53 functions in regulating cell cycle and apoptosis through USP10.

### TP53 regulates miR-138 expression

**Figure 3:**
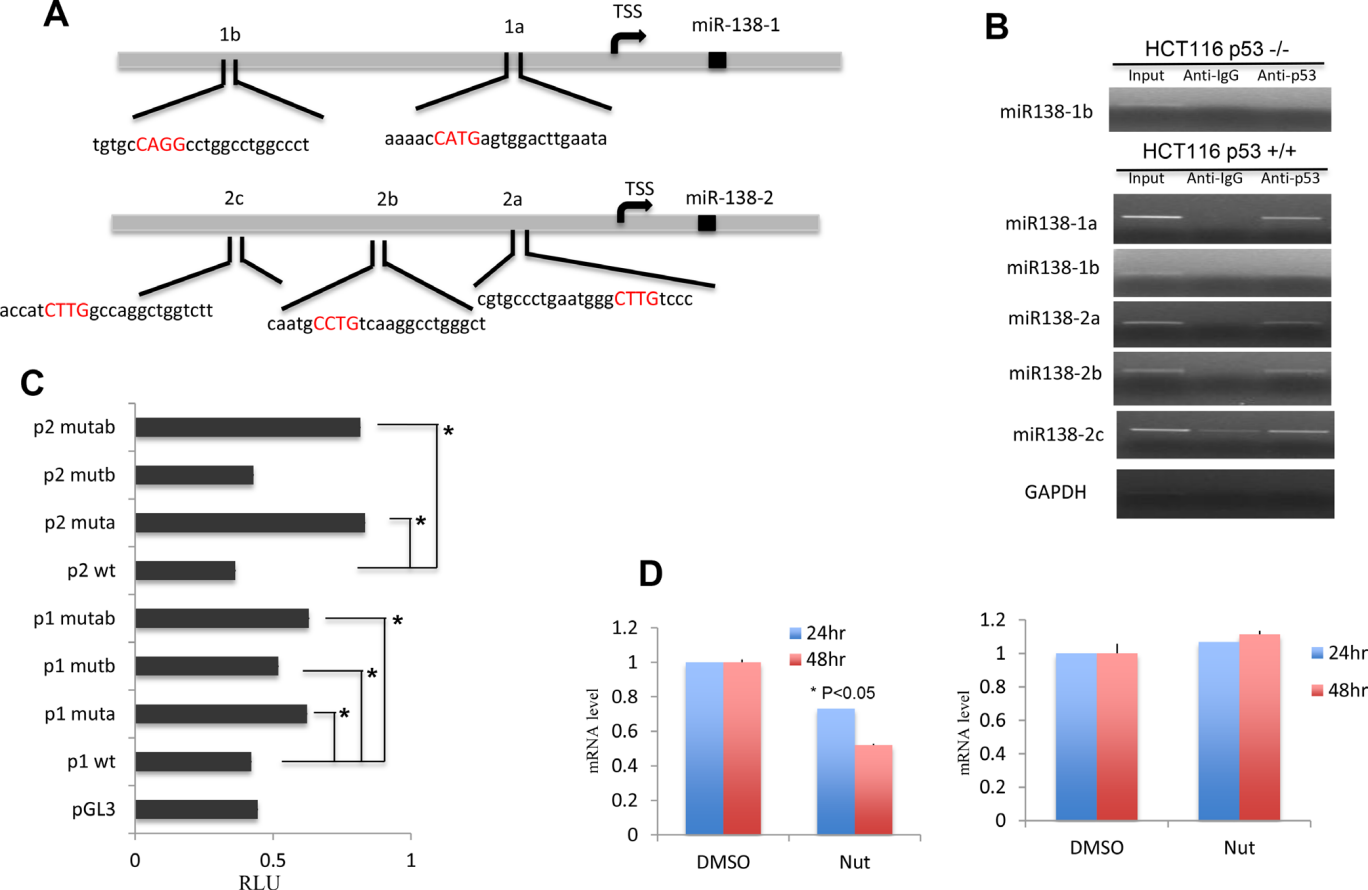
miR-138 expression is regulated by TP53. (**A**) Predicted TP53 binding sites on the miR-138 upstream region. Sites mutated to abrupt TP53 binding were shown in red. (**B**) ChIP analysis to determine TP53 binding. Anti-IgG is used as control to show the specificity of TP53 binding. GAPDH serves as another control to show the specific binding. (**C**) Relative luciferase (RLU) reporter assay to determine the regulatory role of TP53 on miR-138 genes. HeLa cells were co-transfected with TP53 plasmid and pGL3 empty vector control, wild-type, single mutant or double mutant of the upstream sequences of miR-138-1 and miR-138-2. (**D**) Left, miR-138 expression levels measured by Realtime PCR in A549 cells treated with Nutlin-3a or DMSO vehicle. Cells were harvested at 24 hr or 48 hr after the treatment. Right, Realtime PCR results of miR-138 in H1299 cells treated with or without Nutlin-3a at 24 hr and 48 hr.

miR-138 has been widely studied in many cancers. However, how the expression of this important regulator is controlled is largely unknown. In human, there are two genes encoding miR-138, termed miR-138-1 and miR-138-2, located on chromosome 3p21.33 and 16q13, respectively. Interestingly, our *in silico* analyses found there are two and three putative TP53 binding sites on miR-138-1 and miR-138-2, respectively (Figure 3A). To validate whether or not TP53 directly binds to these sites, Chromatin Immunoprecipitation (ChIP) experiments were carried out. As shown in Figure 3B, TP53 antibody successfully pulled down two putative fragments of miR-138-1and two regions of miR-138-2 while the third putative fragment from miR-138-2 gave non-specific binding. As a negative control, we did not see any binding in HCT116 TP53^−/−^ cells. These data suggest TP53 specifically binds to the miR-138 upstream regions.

We then investigated how miR-138 expression is regulated by TP53 with luciferase assays. The upstream regions of miR-138 genes were cloned into pGL3 luciferase vector. Site-directed-mutagenesis was used to disrupt the potential binding of TP53. To our surprise, we found that the luciferase activities were increased when the putative TP53 binding sites were disrupted (Figure 3C), especially when site a in both miR-138-1 and miR-138-2 was disrupted, indicating that TP53 directly regulating miR-138 expression. In order to confirm this finding, we detected miR-138 expression in cells which TP53 activity is enhanced by nutlin-3a. P53 is negatively regulated by mdm2, nutlin-3a is a potent mdm2 inhibitor. In cell line A549 who carries a wild-type TP53, we observed miR-138 mRNA levels were reduced after nutlin-3a treatment, but this effect was not observed in cell line H1299 which carries a TP53 null mutant Figure 3D. These data indicate TP53 could directly bind to miR-138 upstream region and suppresses its expression.

### miR-138 promotes tumor growth *in vivo*

**Figure 4:**
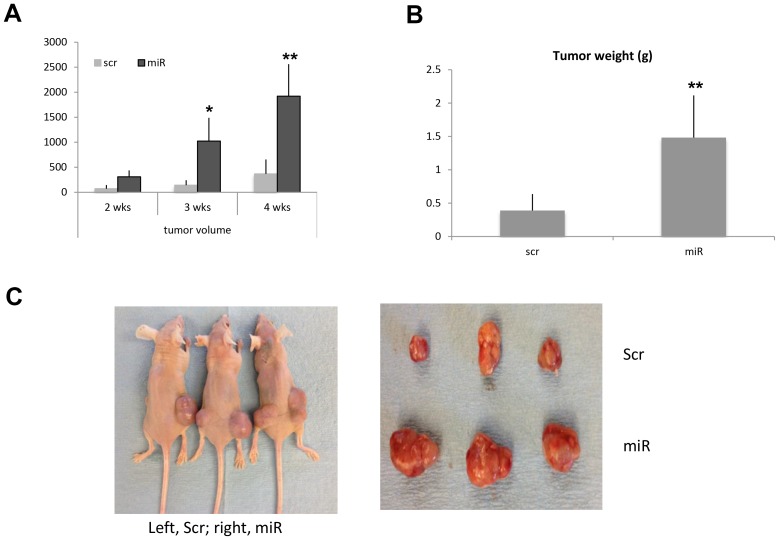
miR-138 promotes tumor growth in nude mice. Two month old nude mice were s.c. injected with one million HeLa cells tranfected with scramble or miR-138 and the tumor volume was measured and calculated as length x width x width/2 at week 2, 3 and 4 after injection (**A**). On week 4, the mice were sacrificed and the tumors were weighted (**B**). (**C**) Pictures were taken at week 4 before the mice sacrificed.

To determine the function of miR-138 *in vivo*, Hela cell line stably expressing miR-138 was established. Hela cells with or without overexpressing miR-138 were injected s. c. into the flanks of nude mice. Started on week 2 after the injection, we observed that either tumor volume or tumor weight was dramatically increased in the side injected with the miR-138 overexpressing cells (*p* < 0.05, *n* = 6. Figure 4). Our data shows overexpression of miR-138 caused substantial tumor growth in nude mice.

## DISCUSSION

**Figure 5:**
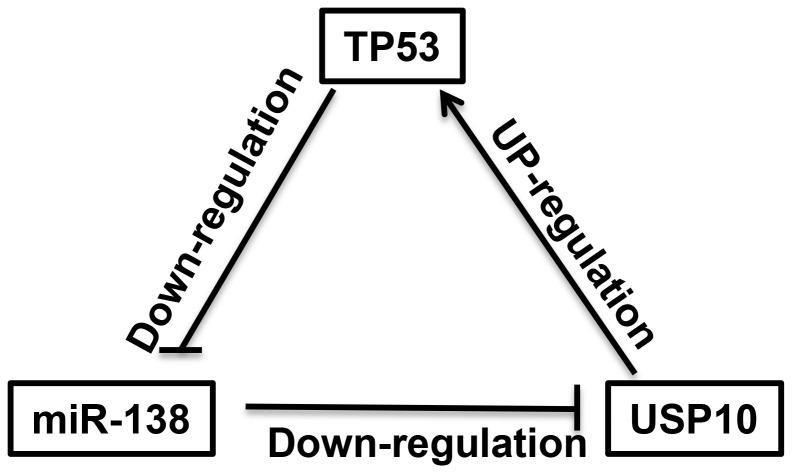
A negative feedback regulatory loop between miR-138 and TP53 is mediated by USP10. miR-138 indirectly suppresses TP53 expression through down-regulation of USP10 whereas p53 directly inhibits miR-138 expression, therefore miR-138 works as an oncogene in cancer cells that carry wild-type p53 while works as a tumor suppressor in cancer cells that carry mutant/null p53.

In approximately 50% of all human cancers, TP53 is either lost or mutated in a way that its function is compromised, resulting in abolished cell senescence and apoptosis. More recently, we know that many of these mutated forms of TP53 gene give rise to mutant TP53 proteins that are highly expressed. There are reports showed that some of the most common mutant TP53 proteins might have acquired a gain of function. They could drive cell migration and metastasis as well as promote tumor growth and progression. Normally, TP53 levels are highly suppressed by an E3 ubiquitin ligase, MDM2. Upon cellular stresses, such as DNA damage or oncogene activation, the TP53 degradation process is attenuated by USP7 and USP10, two deubiquitinating enzymes. Interestingly, USP10 could stabilize both wild-type and mutant TP53 thus functions as either a tumor suppressor or an oncoprotein. In this study, we identified miR-138 as a direct regulator of USP10. Luciferase assays shows miR-138 specifically binds to a conserved region of the USP10 3′-UTR. Overexpressing miR-138 in cells inhibits USP10 mRNA accumulation and protein expression levels. Since USP10 is a positive regulator of TP53, we observed a consistent down-regulation of TP53 mRNA and protein when miR-138 level was increased. Thus, this finding includes miRNA-138 into the TP53 regulation network. We further found miR-138 overexpression inhibits TP53-dependent transcription by repressing USP10 and abrogates TP53-dependent cell apoptosis and cell cycle arrest. We have also identified potential TP53 biding sites in the miR-138 upstream regions. ChIP assays validated that TP53 directly binds to these sequences and thus TP53 could be a regulator of miR-138 expression. Luciferase analysis of these sequences showed TP53 serves as a negative role in miR-138 regulation. When we mutated these putative TP53 biding sites, we found the luciferase activities were increased upon TP53 activation. Furthermore, in A548 cells which carry a wild-type TP53, nultin-3a treatment repressed miR-138 expression, most likely because TP53 activity is stabilized. In contrast, in cell line H1299 who has a null TP53 gene, miR-138 level was not decreased by nutlin-3a treatment. These data indicate miR-138 and TP53 form a negative feedback regulatory loop (Figure 5).

MiR-138 has been investigated in a number of human cancers, including HCC, HNSCC, and gliomas. Interestingly, miR-138 was found to be down-regulated in HCC and HNSCC, indicating that it might serve as a tumor suppressor, whereas in gliomas, miR-138 was suggested to be an oncomiR. Such dual players of the same miRNA in different cancers are not rare. For example, miR-221/222 was recognized as oncogenes in breast cancer, prostate cancer and HCC [29], but it showed tumor suppressor activities in oral tongue squamous cell carcinoma (OTSCC) [30]. In this study, we demonstrated miR-138 is involved in TP53 regulation through its direct targeting USP10. Since USP10 could stabilize both wild-type and mutant TP53 protein, USP10 is regarded as either a tumor suppressor or oncoprotein, depending on the TP53 status. Therefore, miR-138 could also be a tumor suppressor or oncomiR in different cell context (Figure 5). This may explain previous findings that miR-138 is a tumor suppressor in HCC and HNSCC, but it had the ability to promote gliomas malignancy. Another possibility is that miR-138 has different targets in different cancers. Further studies to link the role of miR-138 in tumorigenesis and the status of TP53 would be extremely interesting. miR-138 could be a promising therapeutic tool for the roughly half of human cancers with a mutated/null TP53.

## MATERIALS AND METHODS

### Plasmids and siRNAs

To validate the putative targeting site of miR-138, the 3′ UTR of USP10 gene was amplified by PCR and cloned into pGL3 vector (Promega). To examine the TP53 binding sites, the upstream region of miR-138-1 and miR-138-2 were amplified by PCR and cloned into pGL3 vector. Mutations were generated with the QuickChange Mutagenesis kit (Strategene). All constructs were sequenced to verify accuracy.

### Cell culture and transfection

Human cancer cell lines HCT116, HeLa, A548, H1299 were cultured in RPMI 1640 medium plus 10% heat-inactivated FBS and 100 U/ml penicillin-streptomycin. Transfection of miRNA precursors, miR inhibitors, and siRNAs were performed with Lipofectamine 2000, according to the manufacturer’s instructions (Invitrogen).

### Luciferase assay

Cells were seeded in 24 well plates for 24 hr before transfection. Plasmids constructed in pGL4-luc2 vector were co-transfected with control Renilla luciferase plasmid (pGL4-hRluc/TK) in 10:1 ratio. Luciferase assays were performed using the Dual-Luciferase Receptor Assay System (Promega). In brief, 24-48 hr after transfection, cell lysates were prepared with 1x passive lysis buffer for 15 min at room temperature. Cell lysates were transferred in triplicate to 96-well plates and analyzed with GloMax Luminometer (Promega) following the manufacturer’s instructions.

### Immunofluorescence

Cells were plated on glass coverslips and transfected with miR-138 precursor or scramble control. 48 hr after transfection, cells were fixed, washed and stained with indicated antibodies with the Alexa Fluor SFX Kit from Invitrogen, according to the manufacturer’s instructions.

### Quantitative real-time (q-RT) PCR

Total RNA was prepared from cultured cells using TRizol (Invitrogen) in accordance with manufacture’s instructions. Total RNA was subjected to qRT-PCR. Taqman miRNA assays and gene expression assays were used to analyze mature miRNAs and mRNAs, respectively. RNA concentrations were determined by NanoDrop (NanoDrop Technologies, Inc.). RNU44 or RNU48 was used to normalize miRNA expression. GAPDH or β-actin served as internal control for mRNA quantification. Gene expression levels were quantified with the ABI Prism 7900HT sequence detection system (Applied Biosystems). All these quantification experiments were performed in triplicates, including controls. Relative expression was calculated with the comparative Ct method.

### TP53 binding sites prediction and ChIP assay

Putative TP53 binding sites in the miR-138 promoter regions were predicted by MatInspector (Cartharius *et al*., 2005; Genomatrix). HCT116 TP53^+/+^ and HCT116 TP53 ^−/−^ cells were treated with DMSO or 10um Nutli-3a for 24 hr then cross-linking with 1% formaldehyde for 10 min. Simple ChIP enzymatic chromatin IP kit (Cell Signaling Technology) was used for ChIP assay according to the manufacture’s instructions. 5 ug of anti-TP53 antibody (Santa Cruz) was used to pull down the DNA-protein complexes. Rabbit IgG was used as negative control. The precipitated DNA fragments were subjected to PCR amplification.

### Cell cycle analysis and apoptosis assay

HCT116 cells were transfected with 100 nM scramble or premiR-138 for 24 hr, then starved in serum-free medium for 48 hr and incubated in 10% FBS-containing medium for another 24 hr. The cells were then fixed in 70% ethanol and stained with 20 ug/ml of propidium iodide (Sigma) in PBS buffer containing 0.05% Triton X-100 and 200 ug/mL of RNase A. Cells were analyzed on FACSCalibur and Cell Quest Pro Software (BD Bioscience). Cell apoptosis assay was carried out with the FITC Annexin V Apoptosis Detection Kit (BD Pharmingen), according to the manufacturer’s protocol.

### Western blot analysis and antibodies

Total proteins were extracted in RIPA buffer [25 mM Tris/HCl (pH7.6), 150 mM NaCl, 1% Nonidet P-40, 1% sodium deoxycholate, 0.1% SDS, 1x phosphoate inhibitor mixture and protease inhibitor mixture], and applied onto NuPAGE 4–12% Bis-Tris gel (Invitrogen). Proteins were transferred to Immuno-Blot PVDF membrane (Bio-Rad). The membrane was then blocked in 5% non-fat dry milk in Tris buffered saline with Tween-20, incubated with the specific primary antibody, washed, probed with secondary antibody IgG conjugated to HRP (Pierce), and developed with SuperSignal chemiluminescent substrate (Pierce). Antibodies against TP53, p21, GAPDH were purchased from Santa Cruz. USP10 was from Bethyl Laboratories.

### *In vivo* xenograft experiments

Two groups of three nude mice each were injected with 1 × 10^6^ HeLa cells transfected with premiR-138 plasmid or scramble miRNA. In brief, tranfected cell were harvested by trypsin treatment, washed with PBS and resuspended in Matrigel/PBS (1:1). One million cells were s.c. injected into the flank of nude mice. Tumor volume was measured by caliper weekly and calculated as length × width Times New Roman width/2. Animal experiments were conducted after approval of the Institutional Animal Care and Use Committee, the Ohio State University.

### Statistical analysis

The results were analyzed using ANOVA and/or two paired student *t*-test. Only *p*-values < 0.05 were considered significant.

### Bioinformatics analysis

USP10 and miR-138 interaction was predicted by specific programs: Targetscan, Pictar, and RNhybrid.Transcription factor binding site of miR-138 was investigated on Genomatix. 1. http://www.targetscan.org/; 2. http://bibiserv.techfak.uni-bielefeld.de/; 3. http://www.genomatix.de/.

## SUPPLEMENTARY MATERIALS


