# Supplementary material for: A negative feedback regulatory loop between miR-138 and TP53 is mediated by USP10
Source: Oncotarget. 2019 Oct 29;10(59):6288–96. doi: 10.18632/oncotarget.27275 (PMC6824876; doi:10.18632/oncotarget.27275)
Supplement: Supplementary file 1 [file oncotarget-10-6288-s001.pdf]

A negative feedback regulatory loop between miR-138 and TP53 is mediated by USP10

SUPPLEMENTARY MATERIALS

```
Human    GUCUCAUUGCUUAGUAGAAUA-AAUCCUGCACCAGCAACAA-CACUUGUAAAUU
Chimp    GUCUCAUUGCUUAGUAGAAUA-AGUCCUGCACCAGCAACAA-CACUUGUAAAUU
Mouse    GUCUGAUCGCGUAGUGGAAUA-AGUCCCGCACCAGCAGC----UCUUGUAAAUU--UGUG-AAAAU
Rat      GUUUGAU-GCGUAGUGGAAUA-AGUCCCGCACCAGCAGC----UCUUGUAAAUU--UGUG-AAAAU
Pig      GUCUCAUUGCGUAGUAGAAUA-AAUCCUGCACCAGCAAC----ACUUGUAAAUU--UGUG-AAAAU
Cow      GUCUAAUUGCAUAGUAGAGUA-AGUCCUGCACCAGCAACAA-CACUUGUAAAUU--UGUG-AAAAU
Dog      GUCUAAUUGCUUAGUAGAAUA-AAUCCUGCACCAGCA---A-CACUUGUAAAUU--UGUG-AAAAU

USP10    5'  ...GUAGAAUAAAUCCUGCACCAGCA...
                          | | | | | | |
miR-138  3'   GCCGGACUAAGUGUUGUGGUCGA
```

Supplementary Figure 1: miR-138 targets USP10. The targeting sequence is highlighted in red which is highly conserved in mammals.
